# Cryopyrin associated periodic syndromes (CAPS): immunological characterization of knock-in mouse model to exploit novel approaches for the modulation of the NLRP3 inflammasome.

**DOI:** 10.1186/1546-0096-13-S1-P200

**Published:** 2015-09-28

**Authors:** A Bertoni, S Carta, E Balza, P Catellani, C Pellecchia, F Penco, F Schena, S Borghini, ML Trotta, C Pastorino, I Ceccherini, A Martini, A Rubartelli, M Gattorno, S Chiesa

**Affiliations:** 1 G.Gaslini Institute, II Pediatric Division, Genova, Italy; 2 University of Genoa, Genova, Italy; 3 IRCCS San Martino-Ist, Unità di Biologia Cellulare, Genova, Italy; 4 G.Gaslini Institute, Molecular Genetics, Genova, Italy

## Question

CAPS are autoinflammatory diseases characterized by recurrent episodes of fever and systemic inflammation, subdivides into three different severity phenotypes (FCAS, MWS, CINCA). These syndromes are caused by mutations of NLRP3 gene coding for an intracellular multiprotein complex that mediates IL-1β processing and secretion. These mutations are gain-of-function, resulting in an inflammasome hyperactivity and IL-1β hypersecretion. We aimed to: increase the knowledge on pathologic consequences of NLRP3 mutations in CAPS patients; understand the molecular and regulatory mechanisms of CAPS disease; identify novel molecular targets for the treatment of cryopyrin/NLRP3 related disorders.

## Methods

We generated a Knock-in (KI) mouse carrying the N475K mutation into the murine NLRP3 gene. This mutation corresponds to the N477K human mutation, associated with a severe CINCA phenotype with neurological complications; phenotypical and immunological characterization of KI has been performed by flow cytometry; IL1β secretion from bone marrow derived dendritic cells (BMDCs) and peritoneal macrophages (PMs) of KI has been evaluated by ELISA.

## Results

NLRP3 KI mice show hair loss, skin rash and reduced survival time compared to wild type mice (WT). Autopsy of KI mice, prematurely dead, revealed splenomegaly and a relevant inflammatory status. We compared IL-1β secretion of inflammatory cells from WT and KI mice. PMs and BMDCs from mutant mice did not secrete mature IL-1β spontaneously. When stimulated with 100 ng/ml of LPS KI cells secreted higher levels of IL-1β than WT cells. The kinetics of IL-1β secretion was much faster in KI cells, reaching the plateau at 3h from exposure to LPS, reproducing the results obtained from monocytes of CAPS patients. As in CAPS monocytes, brief exposure to ATP strongly induced the secretion of IL-1β by LPS-activated WT cells while failed to stimulate further IL-1β secretion by KI mice inflammatory cells. Finally, PMs and BMDCs from KI are more responsive to agonists of TLRs compared to WT cells: LPS at 0.01 ng/ml triggered high levels of IL-1β secretion in KI cells indicating that the presence of the mutation lowers the threshold of activation. Immunological and functional studies of peritoneal cavity are in progress, interestingly we noticed a reduction of B lymphochytes especially in innate-like B1 cells. We are also evaluating neurological aspects of CINCA disease in NLRP3 KI mice.

## Conclusions

The NLRP3 KI mice recapitulates phenotype and functional characteristics of CAPS patients. Thus, this model will provide elucidations in the mechanisms underlying CAPS as well as other inflammasomepathies.

